# Development and validation of a risk prediction model for consciousness disorders in stroke patients in the intensive care unit (ICU): a retrospective study

**DOI:** 10.3389/fmed.2025.1668593

**Published:** 2025-12-29

**Authors:** Gang Fang, Liping Wang, Xinhua Liu, Jinyu Liu, Yongle Pei, Yuxia Qi, Haixia Chang

**Affiliations:** 1 School of Nursing, Xinjiang Medical University, Ürümqi, China; 2 Department of Nursing, The Fifth Affiliated Hospital of Xinjiang Medical University, Ürümqi, China

**Keywords:** consciousness disorders, intensive care unit (ICU), machine learning, risk prediction model, stroke

## Abstract

**Objective:**

We used data from stroke patients in the Medical Information Mart for Intensive Care (MIMIC) database to develop and validate risk prediction models for consciousness disorders in stroke patients using 11 machine learning algorithms. It aims to provide a basis for clinical assessment of consciousness changes in stroke patients.

**Methods:**

Data of 2,434 stroke patients were extracted from the MIMIC-IV database and randomly split into a training set and a validation set at a 7:3 ratio. Multivariate logistic regression was employed to identify independent predictors, and 11 machine learning algorithms were used to construct predictive models for post-stroke consciousness disorders. Calibration curves were applied to validate the calibration performance of the models, while decision curve analysis (DCA) was utilized to evaluate their clinical applicability, ultimately determining the optimal predictive model.

**Results:**

A total of 2,434 ICU stroke patients were included, with 1,706 assigned to the training set and 728 to the validation set. Logistic regression analysis identified four independent predictors (all *p* < 0.001): length of hospital stay (*p* < 0.001, 95% confidence interval [CI]: 1.02–1.06), mechanical ventilation (*p* < 0.001, 95% CI: 0.29–0.72), nasogastric tube (*p* < 0.001, 95% CI: 1.61–3.79), and Sequential Organ Failure Assessment (SOFA) score (p < 0.001, 95% CI: 1.47–1.74). Among the 11 machine learning models, the Light Gradient Boosting Machine (LightGBM) model exhibited the optimal performance across three dimensions: accuracy (area under the curve [AUC] = 0.824 in the training set, AUC = 0.795 in the validation set), stability (consistency between training and validation set results), and probability calibration (Brier score = 0.132 in the training set, Brier score = 0.140 in the validation set). Calibration curves demonstrated excellent agreement between the model’s predictions and ideal values in both datasets, and DCA confirmed its favorable clinical utility.

**Conclusion:**

Multivariate analysis revealed that length of hospital stay, mechanical ventilation, nasogastric tube, and SOFA score are independent predictors of consciousness disorders in ICU stroke patients. The model constructed using the LightGBM algorithm showed the best comprehensive performance and can serve as an intuitive, personalized clinical tool. It assists healthcare providers in the early identification and risk stratification of stroke patients at high risk of consciousness disorders, thereby supporting the timely implementation of interventions to reduce the incidence of complications.

## 1 Introduction

Stroke patients are highly prone to consciousness disorders, which present as lethargy, confusion, agitation, or coma. These conditions can precipitate complications including pressure ulcers, urinary tract infections, aspiration pneumonia, and hypostatic pneumonia—markedly compromising patients’ quality of life and elevating their risk of mortality (1). According to existing literature, 4–38% of stroke patients may suffer from varying degrees of coma, whereas 13–48% exhibit symptoms of confusion or delirium (2). Delayed intervention for post-stroke consciousness disorders can further result in aphasia, dysphagia, and pulmonary infections, which in turn prolong the rehabilitation period, increase medical expenditures, impede functional recovery and prognostic improvement, and even pose life-threatening hazards (3). Notably, stroke patients with altered consciousness typically require admission to the Intensive Care Unit (ICU) for treatment (4), and those with concurrent consciousness disorders have a substantially higher mortality rate compared to patients with normal consciousness (5). Therefore, the timely identification and intervention of consciousness changes in post-stroke patients are crucial for optimizing patient prognosis.

A predictive model refers to a tool that estimates the probability of prognostic events by leveraging clinicopathological parameters (6). While prior studies have examined the influencing factors of post-stroke delirium and established predictive models for this condition (7–10), a critical gap remains: there is an urgent need to utilize multicenter, large-sample datasets and integrate multiple machine learning algorithms to develop an optimal predictive model tailored specifically to post-stroke consciousness disorders.

Jointly developed by the Massachusetts Institute of Technology (MIT) and Beth Israel Deaconess Medical Center (BIDMC), the Medical Information Mart for Intensive Care IV (MIMIC-IV) database houses comprehensive clinical data from nearly 70,000 ICU patients treated at BIDMC between 2008 and 2019. Its core data domains encompass demographic characteristics, vital sign records, laboratory test results, medication regimens, medical device utilization (e.g., mechanical ventilators, nasogastric tubes), International Classification of Diseases (ICD) codes, and in-hospital outcomes (e.g., in-hospital death, discharge location) (11). Owing to its large sample size, diverse data categories, well-structured format, and accessibility for retrieval and analysis, MIMIC-IV has become a widely adopted resource in research focused on critical illnesses such as stroke and sepsis (12).

In this retrospective study, we developed a predictive model for post-stroke Consciousness Disorders by analyzing potential risk factors extracted from the MIMIC-IV database.

## 2 Materials and methods

### 2.1 Data source

A retrospective cohort analysis was performed using data extracted from the MIMIC-IV Version 3.1 database (13). Gang Fang (ID: 69157712), an author of this study, completed the required registration process and all mandatory training modules through the National Institutes of Health (NIH) Collaborative Agreement Online platform. This access was granted following approval by the Institutional Review Board (IRB) of the MIT. Mr. Fang maintains valid authorization to access, retrieve, and validate data within the MIMIC-IV database.

### 2.2 Study population

Inclusion Criteria: Patient data were retrieved from the MIMIC-IV database in accordance with the following criteria: (1) All participants met the diagnostic criteria for ischemic stroke as defined in the Chinese Guidelines for the Diagnosis and Treatment of Acute Ischemic Stroke (2023) (14), or the diagnostic criteria for hemorrhagic stroke as outlined in the Chinese Guidelines for the Diagnosis and Treatment of Intracerebral Hemorrhage (2019) (15); (2) Age ≥ 18 years; (3) Onset of consciousness disorders occurring within 24 h following stroke onset.

Exclusion Criteria: (1) Age < 18 years (*n* = 0); (2) Comorbid conditions that could interfere with the assessment of consciousness disorders (e.g., dementia, pre-stroke consciousness impairment) (*n* = 326); (3) A history of cardiopulmonary resuscitation (*n* = 20); (4) Missing data accounting for more than 20% of total variables (*n* = 2).

**Figure 1:**
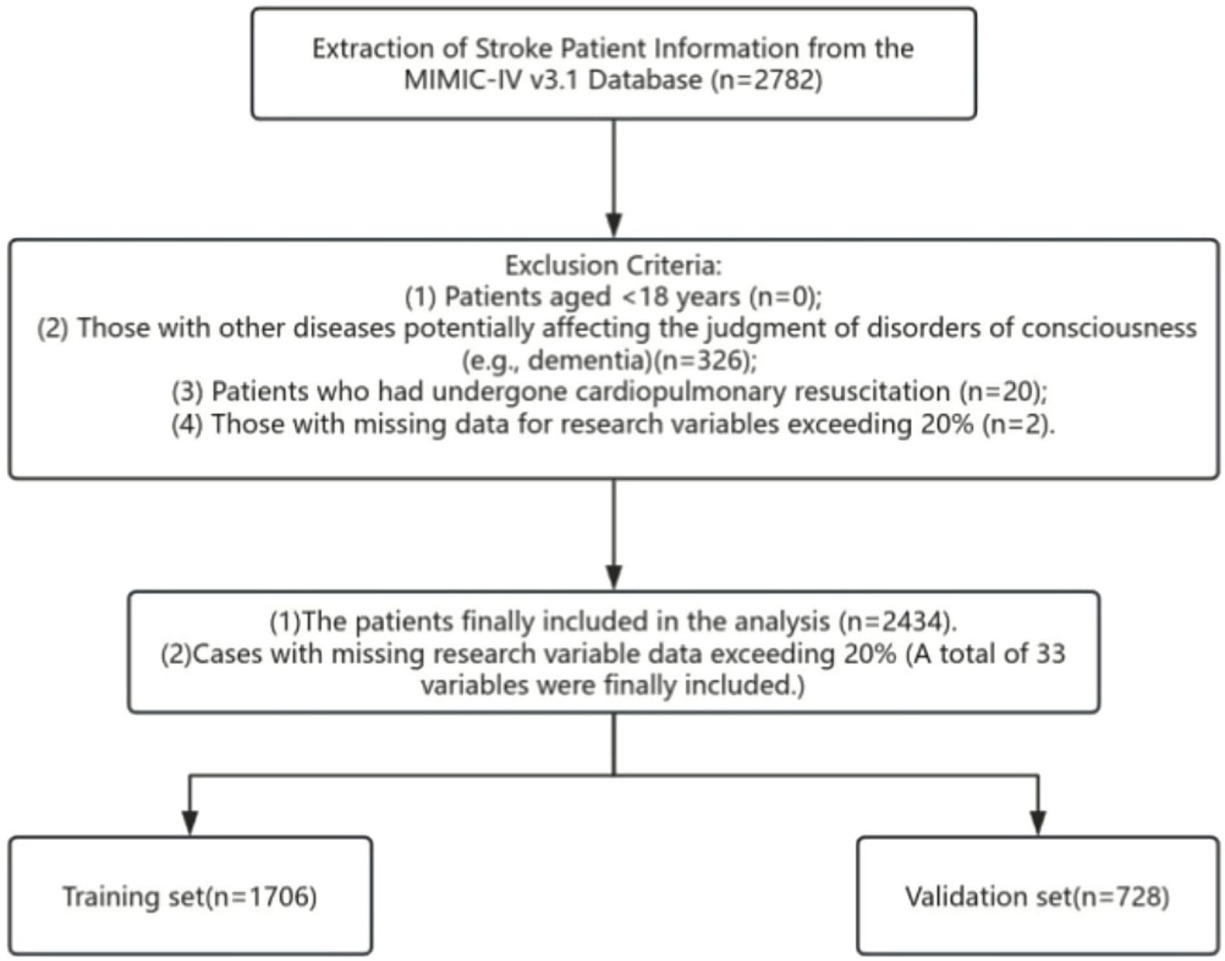
Flow diagram of patient selection.

The patient selection workflow is depicted in Figure 1. Previous studies have established the Glasgow Coma Scale (GCS) as the gold standard for evaluating patients’ level of consciousness (16). In the current study, the GCS was utilized to assess patients’ consciousness status at the time of hospital admission, with consciousness categorized into two distinct groups: conscious and consciousness-impaired. The GCS score spans a range of 3 to 15 points: a score of 15 denotes a fully conscious state, whereas a score below 15 indicates the presence of consciousness disorders (17). Consistent with this classification, patients in the present study with a GCS score < 15 were allocated to the consciousness disorders group, while those with a GCS score = 15 were assigned to the non-consciousness disorders group.

### 2.3 Statistical analysis

Statistical analyses were performed using SPSS 27.0 and R 4.4.3 software. Normally distributed quantitative data were expressed as mean ± standard deviation (SD), while non-normally distributed quantitative data were described using median and interquartile range (IQR). Categorical variables were presented as frequencies or percentages. For intergroup comparisons, the independent-samples *t*-test or Mann–Whitney *U* test was used for continuous variables, and the chi-square test or Fisher’s exact test was applied for categorical variables. Variables with a missing value rate exceeding 20% were directly excluded. For variables with a missing value rate of ≤20%—including fasting blood glucose, potassium, and sodium—simple mean imputation was applied to address missing values. The dataset was randomly divided into a training set and a validation set at a 7:3 ratio.

Univariate and multivariate logistic regression analyses were used to identify risk factors associated with post-stroke consciousness disorders, which informed the construction of the predictive model. Receiver operating characteristic (ROC) curves were employed to evaluate model performance, with the area under the curve (AUC) calculated to quantify the diagnostic efficacy of the models. Calibration curves were utilized to verify the calibration of the models, while decision curve analysis (DCA) was performed to assess their clinical utility. Detailed procedures for generating the DCA curves are provided in Supplementary Material S1. The level of statistical significance was set at *p* < 0.05.

Eleven machine learning algorithms were employed for model construction and validation, encompassing Decision Tree (DT), Ridge Regression, Elastic Net (ENet), Logistic Regression (LR), Least Absolute Shrinkage and Selection Operator (LASSO) Regression, Random Forest (RF), eXtreme Gradient Boosting (XGBoost), Gradient Boosting Machine (GBM), K-Nearest Neighbors (KNN), Support Vector Machine (SVM), and Multi-Layer Perceptron (MLP). Five-fold cross-validation and hyperparameter tuning were implemented to optimize model performance.

Evaluation metrics included the Area Under the Receiver Operating Characteristic Curve (AUC), accuracy, F1-score, and Brier score, all of which were utilized to comprehensively assess the predictive capability of each model. Calibration curves and Decision Curve Analysis (DCA) were separately applied to verify the calibration effectiveness and clinical applicability of the models, ultimately identifying the optimal one.

SHAP (SHapley Additive exPlanations) is a game-theoretic approach designed to interpret the outputs of machine learning models. By leveraging the classic Shapley value from game theory and its relevant extensions, it integrates optimal contribution allocation with local interpretations. In essence, SHAP values can quantify the contribution of each feature to the individual prediction results of a single sample as well as the overall model output. This enables us to conduct a clear and consistent ranking of feature importance based on their marginal impacts.

### 2.4 Machine learning model training and hyperparameter optimization

Regarding the LightGBM model, the specific parameters employed were as follows: the number of boosting iterations (n_estimators) was set to 1,000; the learning rate was 0.05; the maximum depth (max_depth) was configured to 7; and the number of leaf nodes (num_leaves) was restricted to 31. Importantly, an early stopping criterion (with an early stopping tolerance of 50 rounds) was implemented on a held-out validation set (accounting for 20% of the training data) to mitigate model overfitting and determine the optimal number of boosting rounds. The model that achieved the best performance on this validation set was selected as the final model for subsequent evaluations. Furthermore, the complete parameters of all other models (e.g., Random Forest, Support Vector Machine, Logistic Regression) have been tabulated in Table 1. These detailed specifications enable other researchers to precisely replicate our model training process, thereby enhancing the scientific rigor and credibility of the study findings (see Table 1 for additional details).

**Table 1:** Relevant parameters of various machine learning models.

Model	Engine	Core hyperparameters and hyperparameter tuning	Optimal hyperparameters	Final training parameters
DT	rpart (CART)	Max depth	7	7
		Min split/min node size	33	33
		Min bucket	—	11
		Complexity Param (CP)	0.0000171	0.266515
		Split criterion	—	gini
		Cross-validation	—	x-fold
		Early stopping	—	None (via CP pruning)
RF	randomForest	Number of trees	792	792
		Mtry	2	2
		Min node size	55	—
		Max depth	—	None (full depth)
		Split criterion	—	gini (Mean Decrease Gini)
		OOB score	—	0.1952
		Early stopping	—	None (fixed ntree)
XGBoost	xgboost	Max depth	12	12
		Learning rate	0.0613	0.06132
		Min child weight	40	40
		Gamma/loss reduction	0.0000055	0
		Subsample	0.89	0.89
		Colsample_bytree	—	1
		Number of trees	—	284
		Objective	—	binary:logistic
		Early stopping	—	25 rounds
LightGBM	lightgbm	Max depth	2	2
		Learning rate	0.00439	0.00439
		Min data in leaf	24	24
		Feature fraction	—	0.75
		Bagging fraction	—	1
		Number of trees	1,286	1,286
		Objective	—	binary
SVM	kernlab (RBF-SVM)	Kernel	—	rbfdot
		Sigma	6.14E-09	0
		Cost (C)	0.171	0.170702
		Class weights	—	Default (all = 1)
		Support vectors	—	661
MLP	nnet	Hidden units	7	7
		Penalty (Decay)	0.000398	0.000398
		Epochs (MaxIter)	152	152
		Input nodes	—	4
		Hidden nodes	—	7
		Output nodes	—	1
KNN	kknn	Neighbors (k)	35	35
		Weight function/Kernel	triangular	epanechnikov
		Distance power	—	2
		Regression	—	No
		Probabilities	—	Yes
		Predictors	—	4
		Training samples	—	1706
Logistic	glm	Coefficients	—	(Intercept) = −0.615, Hospital length of stay = 0.0393, Mechanical ventilation = −0.8715, Nasogastric tube = 1.011, SOFA score = 0.347
		Predictors	—	4
		Training Samples	—	1706
LASSO	glmnet	Mixture	1 (LASSO)	1
		Penalty (lambda)	0.0151	0.0151
		Coefficients	—	(Intercept) = 1.33, Hospital length of stay = 0.247, Nasogastric tube = 0.223, SOFA score = 0.852
Ridge	glmnet	Mixture	0 (Ridge)	0
		Penalty (lambda)	0.5175	0.5175
		Coefficients	—	(Intercept) = 1.10, Hospital length of stay = 0.0992, Mechanical ventilation = 0.0738, Nasogastric tube = 0.137, SOFA score = 0.186
ENet	glmnet	Mixture (alpha)	0.0565	0.0565
		Penalty (lambda)	0.6699	0.6699
		Coefficients	—	(Intercept) = 1.07, Hospital length of stay = 0.0450, Mechanical ventilation = 0.0341, Nasogastric tube = 0.0875, SOFA score = 0.125

AUC, area under the ROC curve; DT, decision tree; ENet, elastic net; KNN, K-nearest neighbors; Lightgbm, light gradient boosting machine; RF, random forest; Xgboost, eXtreme gradient boosting; SVM, support vector machine; MLP, multi-layer perceptron.

## 3 Results

### 3.1 General characteristics

A total of 2,434 ICU stroke patients were included in this study, among whom 1,802 (74.0%) developed consciousness disorders within 24 h of stroke onset. Table 2 presents the demographic and clinical baseline characteristics of the patients stratified by consciousness status, while Table 3 provides detailed results of univariate analysis—identifying variables with statistically significant differences between the consciousness disorder group and non-consciousness disorder group (all *p* < 0.05).

**Table 2:** The characteristics of patients included in the training set (stratified by risk of altered consciousness).

Name	Levels	Non-consciousness disorders (*N* = 439)	Consciousness disorders (*N* = 1,267)	*p*-value
Gender	Male	222 (50.6%)	655 (51.7%)	0.725
	Female	217 (49.4%)	612 (48.3%)	
Age	Mean ± SD	69.3 ± 14.8	69.3 ± 15.7	0.923
Hospital length of stay	Mean ± SD	6.7 ± 10.1	12.6 ± 13.2	<0.001
Smoke history	0	404 (92%)	1,190 (93.9%)	0.204
	1	35 (8%)	77 (6.1%)	
Alcohol abuse	0	437 (99.5%)	1,259 (99.4%)	0.958
	1	2 (0.5%)	8 (0.6%)	
Hypertension	0	196 (44.6%)	551 (43.5%)	0.715
	1	243 (55.4%)	716 (56.5%)	
Diabetes	0	279 (63.6%)	878 (69.3%)	0.031
	1	160 (36.4%)	389 (30.7%)	
Heart failure	0	339 (77.2%)	1,004 (79.2%)	0.410
	1	100 (22.8%)	263 (20.8%)	
Liver disease	0	435 (99.1%)	1,253 (98.9%)	0.943
	1	4 (0.9%)	14 (1.1%)	
Renal disease	0	354 (80.6%)	1,052 (83%)	0.288
	1	85 (19.4%)	215 (17%)	
Pulmonary disease	0	346 (78.8%)	1,007 (79.5%)	0.820
	1	93 (21.2%)	260 (20.5%)	
Cerebrovascular disease	0	53 (12.1%)	74 (5.8%)	<0.001
	1	386 (87.9%)	1,193 (94.2%)	
Malignant cancer	0	400 (91.1%)	1,191 (94%)	0.049
	1	39 (8.9%)	76 (6%)	
Mechanical ventilation	0	353 (80.4%)	722 (57%)	<0.001
	1	86 (19.6%)	545 (43%)	
Antibiotic	0	311 (70.8%)	722 (57%)	<0.001
	1	128 (29.2%)	545 (43%)	
Urinary catheter	0	367 (83.6%)	921 (72.7%)	<0.001
	1	72 (16.4%)	346 (27.3%)	
Nasogastric tube	0	350 (79.7%)	584 (46.1%)	<0.001
	1	89 (20.3%)	683 (53.9%)	
Simplified acute physiology score II (SAPSII)	Mean ± SD	29.9 ± 10.5	35.7 ± 13.3	<0.001
Sequential organ failure assessment (SOFA)score	Mean ± SD	2.5 ± 2.4	5.0 ± 3.1	<0.001
Temperature	Mean ± SD	36.6 ± 0.7	36.7 ± 0.8	0.006
Heart rate	Mean ± SD	83.0 ± 18.9	83.5 ± 17.6	0.601
Resp rate	Mean ± SD	17.8 ± 4.9	18.5 ± 5.4	0.014
Systolic blood pressure	Mean ± SD	132.3 ± 22.4	133.3 ± 25.7	0.401
White blood cell	Mean ± SD	11.1 ± 11.8	11.6 ± 5.7	0.444
Platelet	Mean ± SD	207.9 ± 75.0	217.8 ± 90.7	0.024
Red blood cell	Mean ± SD	3.8 ± 0.7	3.8 ± 0.8	0.925
Creatinine	Mean ± SD	1.1 ± 0.8	1.2 ± 1.3	0.180
Blood urea nitrogen	Mean ± SD	21.2 ± 14.6	22.4 ± 18.9	0.161
Prothrombin time	Mean ± SD	14.7 ± 7.9	14.9 ± 7.9	0.715
Fasting blood glucose	Mean ± SD	133.4 ± 39.5	137.9 ± 44.1	0.050
Potassium	Mean ± SD	4.1 ± 0.7	4.0 ± 0.7	0.027
Sodium	Mean ± SD	138.3 ± 4.1	138.9 ± 4.9	0.009
Hemoglobin	Mean ± SD	11.8 ± 2.0	11.8 ± 2.1	0.791

Red-highlighted sections: data with statistical significance.

Data were expressed as mean ± SD for continuous variables and percentage for categorical variables. SD, standard deviation.

**Table 3:** Univariate and multivariate logistic analysis was performed on the training group.

Name	Level	OR (univariable)	OR (multivariable)
Gender	Male		
	Female	0.96 (0.77–1.19, *p* = 0.684)	
Age	Mean ± SD	1.00 (0.99–1.01, *p* = 0.923)	
Hospital length of stay	Mean ± SD	1.10 (1.08–1.12, *p* < 0.001)	1.04 (1.02–1.06, *p* < 0.001)
Smoke history	No		
	Yes	0.75 (0.49–1.13, *p* = 0.168)	
Alcohol abuse	No		
	Yes	1.39 (0.29–6.56, *p* = 0.679)	
Hypertension	No		
	Yes	1.05 (0.84–1.30, *p* = 0.673)	
Diabetes	No		
	Yes	0.77 (0.62–0.97, *p* = 0.027)	
Heart failure	No		
	Yes	0.89 (0.68–1.15, *p* = 0.373)	
Liver disease	No		
	Yes	1.22 (0.40–3.71, *p* = 0.732)	
Renal disease	No		
	Yes	0.85 (0.64–1.12, *p* = 0.257)	
Pulmonary disease	No		
	Yes	0.96 (0.74–1.25, *p* = 0.767)	
Cerebrovascular disease	No		
	Yes	2.21 (1.53–3.21, *p* < 0.001)	1.30 (0.85–1.98, *p* = 0.232)
Malignant cancer	No		
	Yes	0.65 (0.44–0.98, *p* = 0.039)	0.53 (0.33–0.86, *p* = 0.011)
Mechanical ventilation	No		
	Yes	3.10 (2.39–4.02, *p* < 0.001)	0.45 (0.29–0.72, *p* < 0.001)
Antibiotic	No		
	Yes	1.83 (1.45–2.32, *p* < 0.001)	0.68 (0.50–0.91, *p* = 0.011)
Urinary catheter	No		
	Yes	1.91 (1.45–2.54, *p* < 0.001)	1.37 (0.99–1.90, *p* = 0.061)
Nasogastric tube	No		
	Yes	4.60 (3.55–5.95, *p* < 0.001)	2.47 (1.61–3.79, *p* < 0.001)
Simplified acute physiology score II (SAPSII)	Mean ± SD	1.04 (1.03–1.05, *p* < 0.001)	0.99 (0.98–1.01, *p* = 0.345)
Sequential organ failure assessment (SOFA) score	Mean ± SD	1.50 (1.42–1.59, *p* < 0.001)	1.60 (1.47–1.74, *p* < 0.001)
Temperature	Mean ± SD	1.20 (1.04–1.38, *p* = 0.011)	
Heart rate	Mean ± SD	1.00 (1.00–1.01, *p* = 0.601)	
Resp rate	Mean ± SD	1.03 (1.00–1.05, *p* = 0.018)	
Systolic blood pressure	Mean ± SD	1.00 (1.00–1.01, *p* = 0.431)	
White blood cell	Mean ± SD	1.01 (0.99–1.03, *p* = 0.295)	
Platelet	Mean ± SD	1.00 (1.00–1.00, *p* = 0.040)	
Red blood cell	Mean ± SD	1.01 (0.87–1.16, *p* = 0.925)	
Creatinine	Mean ± SD	1.06 (0.96–1.17, *p* = 0.277)	
Blood urea nitrogen	Mean ± SD	1.00 (1.00–1.01, *p* = 0.217)	
Prothrombin time	Mean ± SD	1.00 (0.99–1.02, *p* = 0.716)	
Fasting blood glucose	Mean ± SD	1.00 (1.00–1.01, *p* = 0.063)	
Potassium	Mean ± SD	0.84 (0.71–0.98, *p* = 0.028)	
Sodium	Mean ± SD	1.03 (1.01–1.05, *p* = 0.018)	
Hemoglobin	Mean ± SD	0.99 (0.94–1.05, *p* = 0.791)	

Red-highlighted sections: data with statistical significance.

Univariate analysis revealed distinct clinical profiles between the two groups:

Comorbidity-related variables: Diabetes was less frequent in the consciousness-disorder group, whereas cerebrovascular disease was substantially more prevalent in this group; malignant tumor was less common in patients with consciousness disorders.

Supportive intervention variables: Mechanical ventilation, antibiotic use, urinary catheterization, and nasogastric tube placement were all significantly more frequent in the consciousness-disorder group, with nasogastric tube use and mechanical ventilation showing the strongest univariate associations with altered consciousness.

Physiological and laboratory variables: Patients with consciousness disorders had longer hospital stays, higher Simplified Acute Physiology Score II (SAPS II) and Sequential Organ Failure Assessment (SOFA) scores, slightly higher body temperatures, and higher respiratory rates. Laboratory findings indicated that platelet counts and serum sodium levels were higher, while serum potassium levels were lower in the consciousness-disorder group compared to the non-consciousness-disorder group.

Specific variables with significant univariate differences (all *p* < 0.05) are listed in Table 3, including hospital length of stay, diabetes, cerebrovascular disease, malignant tumor, mechanical ventilation, antibiotic use, urinary catheterization, nasogastric tube, SAPS II score, SOFA score, body temperature, respiratory rate, platelet count, serum potassium level, and serum sodium level.

### 3.2 Multivariate logistic regression analysis

Multivariate logistic regression analysis, adjusted for potential confounders, confirmed four variables as independent predictors of consciousness disorders in ICU stroke patients (all *p* < 0.001; Table 3):

(1) Mechanical ventilation (OR = 0.45; 95% CI: 0.29–0.72);
(2) Hospital length of stay (OR = 1.04; 95% CI: 1.02–1.06);
(3) Nasogastric tube (OR = 2.47; 95% CI: 1.61–3.79);
(4) SOFA score (OR = 1.60; 95% CI: 1.47–1.74);

### 3.3 Development and validation of machine learning prediction models

Four variables with *p* < 0.001 from the multivariate analysis (length of hospital stay, mechanical ventilation, nasogastric tube, and SOFA score) were used to compare the performance of 11 machine learning models in the training and test sets. Evaluation metrics included AUC, accuracy, sensitivity, specificity, F1 score, and Brier score (Table 4).

**Table 4:** Comparison of performance metrics among different machine learning models.

Training set							Validation set						
Model	AUC	Accuracy	Sensitivity	Specificity	F1 Score	Brier score	Model	AUC	Accuracy	Sensitivity	Specificity	F1 Score	Brier score
Logistic	0.788	0.687	0.666	0.747	0.76	0.151	Logistic	0.776	0.658	0.637	0.715	0.733	0.157
DT	0.828	0.8	0.863	0.617	0.865	0.126	DT	0.779	0.772	0.852	0.549	0.846	0.145
LASSO	0.78	0.709	0.715	0.69	0.785	0.156	LASSO	0.764	0.685	0.688	0.679	0.763	0.162
Ridge	0.772	0.773	0.861	0.517	0.849	0.173	Ridge	0.745	0.773	0.867	0.513	0.849	0.179
ENet	0.773	0.788	0.892	0.49	0.862	0.179	ENet	0.745	0.786	0.895	0.482	0.86	0.184
KNN	0.857	0.727	0.686	0.845	0.789	0.126	KNN	0.77	0.648	0.619	0.731	0.721	0.146
Lightgbm	0.824	0.77	0.803	0.672	0.838	0.132	Lightgbm	0.795	0.755	0.796	0.642	0.827	0.14
RF	0.9	0.787	0.747	0.9	0.839	0.147	RF	0.773	0.691	0.688	0.699	0.766	0.164
Xgboost	0.82	0.774	0.815	0.658	0.843	0.137	Xgboost	0.785	0.754	0.802	0.622	0.827	0.146
SVM	0.78	0.709	0.723	0.67	0.787	0.191	SVM	0.755	0.681	0.692	0.653	0.761	0.195
MLP	0.812	0.775	0.826	0.631	0.845	0.166	MLP	0.785	0.762	0.821	0.601	0.835	0.171

Yellow-highlighted sections: the optimal model.

AUC, area under the ROC Curve; DT, decision tree; ENet, elastic net; KNN, K-nearest neighbors; Lightgbm, light gradient boosting machine; RF, random forest; Xgboost, eXtreme gradient boosting; SVM, support vector machine; MLP, multi-layer perceptron.

#### 3.3.1 Performance on the training set

AUC: The Random Forest (RF) model performed optimally (0.900), significantly outperforming other models; K-Nearest Neighbors (KNN) (0.857) and Decision Tree (DT) (0.828) followed in sequence.

Accuracy: The Elastic Net (ENet) model achieved the highest accuracy (0.788), closely followed by RF (0.787) and Multi-Layer Perceptron (MLP) (0.775).

Sensitivity: The ENet model exhibited the best sensitivity (0.892), with Ridge Regression (0.861) and DT (0.863) reaching comparable levels.

Specificity: The RF model had the highest specificity (0.900), with KNN (0.845) ranking second.

F1 Score: The DT model obtained the highest F1 Score (0.865), while ENet (0.862) and XGBoost (0.843) also performed well.

Brier Score: The DT and KNN models tied for the lowest Brier Score (0.126), indicating the highest accuracy in probability prediction; SVM had the highest Brier Score (0.191).

#### 3.3.2 Performance on the validation set

AUC: The LightGBM model performed optimally (0.795), followed by XGBoost (0.785) and DT (0.779).

Accuracy: The ENet model achieved the highest accuracy (0.786), with DT (0.772) and MLP (0.762) showing stable performance.

Sensitivity: The ENet model remained the top performer in sensitivity (0.895), while Ridge Regression (0.867) and MLP (0.821) maintained high levels.

Specificity: The KNN model had the highest specificity (0.731), followed by Logistic Regression (0.715).

F1 Score: The MLP model obtained the highest F1 Score (0.835), with LightGBM and XGBoost (both 0.827) showing stable performance.

Brier Score: The LightGBM model had the lowest Brier Score (0.140), indicating the highest accuracy in probability prediction; SVM still had the highest Brier Score (0.195).

#### 3.3.3 Key conclusions

**Figure 2:**
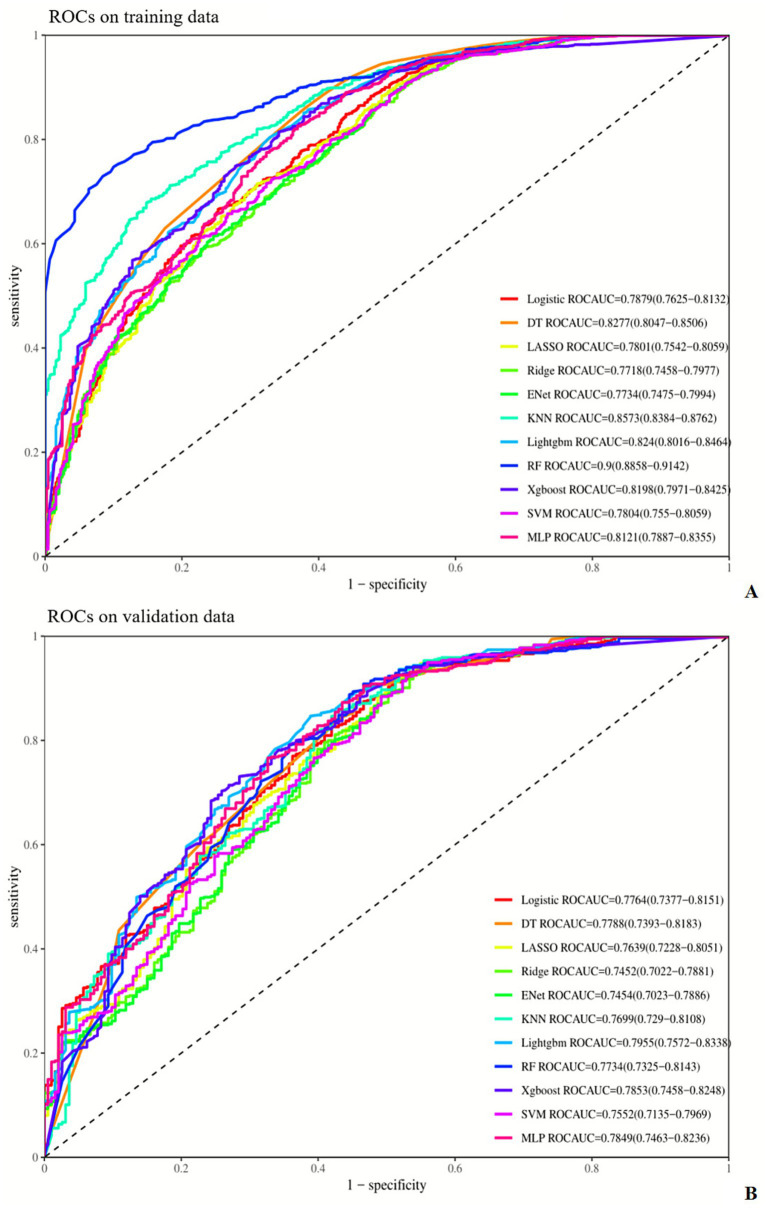
Comparison of ROC curves of different machine learning models in the training **(A)** and validation **(B)** sets. AUC, area under the ROC curve; DT, decision tree; ENet, elastic net; KNN, K-nearest neighbors; Lightgbm, light gradient boosting machine; RF, random forest; Xgboost, eXtreme gradient boosting; SVM, support vector machine; MLP, multi-layer perceptron.

**Figure 3:**
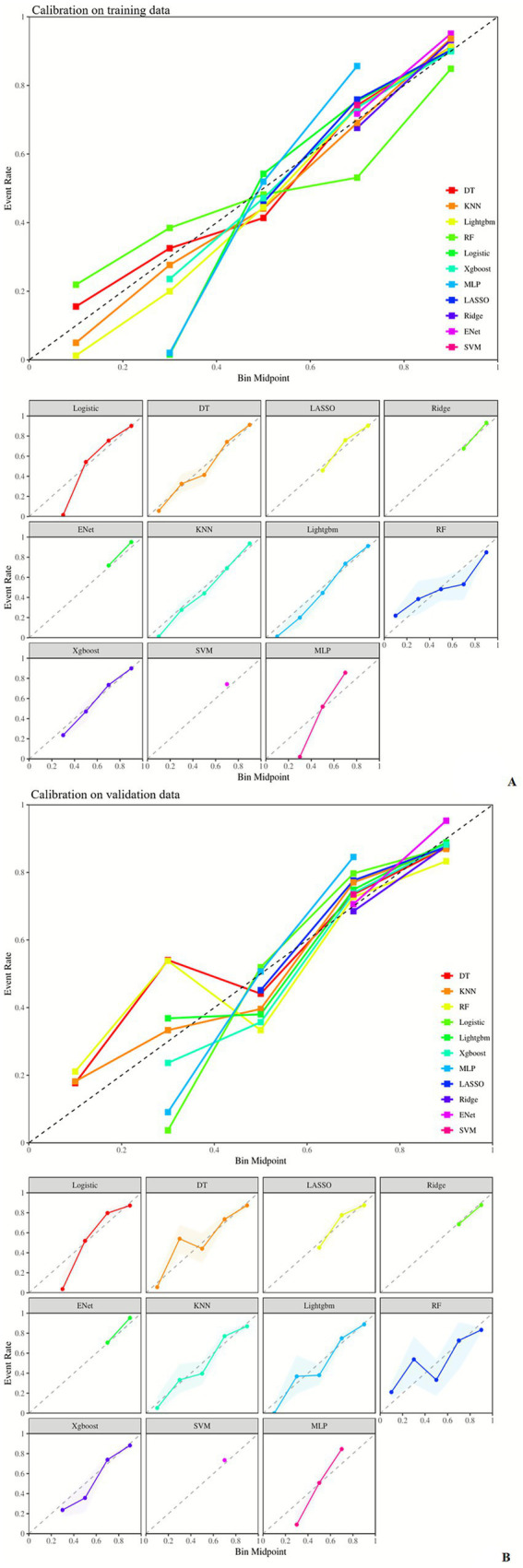
Calibration curves of different machine learning models in the training set **(A)** and validation set **(B)**. AUC, area under the ROC curve; DT, decision tree; ENet, elastic net; KNN, K-nearest neighbors; Lightgbm, light gradient boosting machine; RF, random forest; Xgboost, eXtreme gradient boosting; SVM, support vector machine; MLP, multi-layer perceptron.

**Figure 4:**
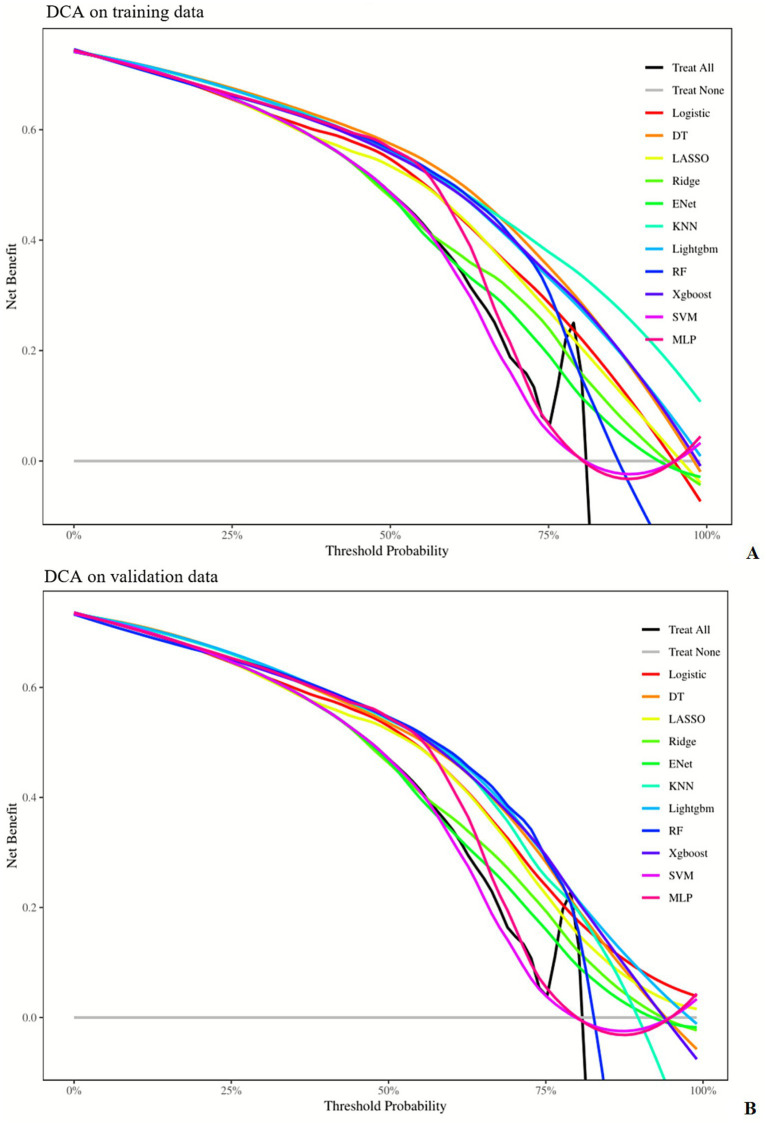
Decision curve analysis (DCA) curves of different machine learning models in the training set **(A)** and validation set **(B)**. AUC, area under the ROC curve; DT, decision tree; ENet, elastic net; KNN, K-nearest neighbors; Lightgbm, light gradient boosting machine; RF, random forest; Xgboost, eXtreme gradient boosting; SVM, support vector machine; MLP, multi-layer perceptron.

**Figure 5:**
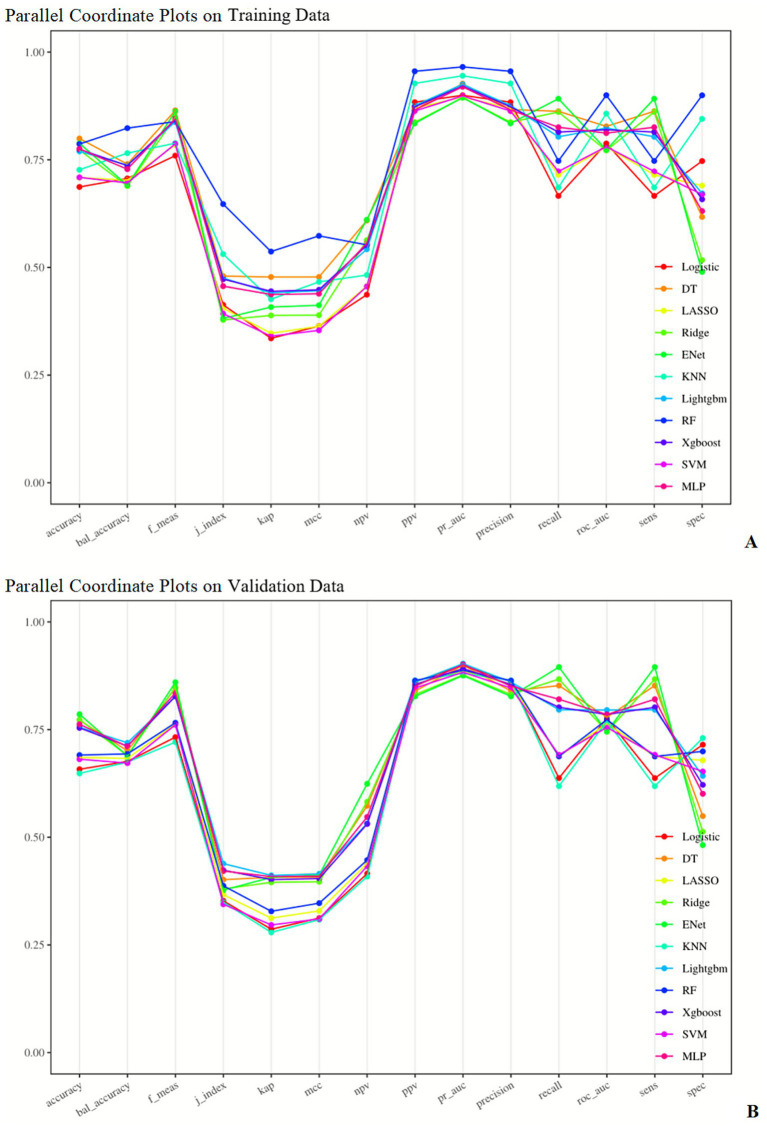
Parallel coordinate plots of different machine learning models in the training set **(A)** and validation set **(B)**. AUC, area under the ROC curve; DT, decision tree; ENet, elastic net; KNN, K-nearest neighbors; Lightgbm, light gradient boosting machine; RF, random forest; Xgboost, eXtreme gradient boosting; SVM, support vector machine; MLP, multi-layer perceptron.

The RF model exhibited the best comprehensive performance on the training set, but its AUC dropped to 0.773 on the test set—suggesting a tendency toward overfitting. The LightGBM model achieved the highest AUC (0.795) and the lowest Brier Score (0.140) on the test set, demonstrating optimal generalization ability and stability in probability prediction. The ENet model showed outstanding performance in sensitivity (0.892 in the training set, 0.895 in the test set) but the lowest specificity (0.490 in the training set, 0.482 in the test set), indicating that the improvement in sensitivity came at the cost of specificity. The SVM model had the highest Brier Score in both sets, indicating the poorest performance in probability prediction. Details are shown in Figures 2–5 and Table 4.

Our Decision Curve Analysis (DCA) results demonstrated that the model yields positive net benefits within a clinically relevant risk threshold range of approximately 30 to 90%. This indicates that within this probability threshold, utilizing our model to guide clinical decision-making—such as initiating specific interventions or further diagnostic evaluations—can lead to superior patient outcomes compared to the strategies of “treating all patients” or “treating no patients,” as detailed in Figure 4.

**Figure 6:**
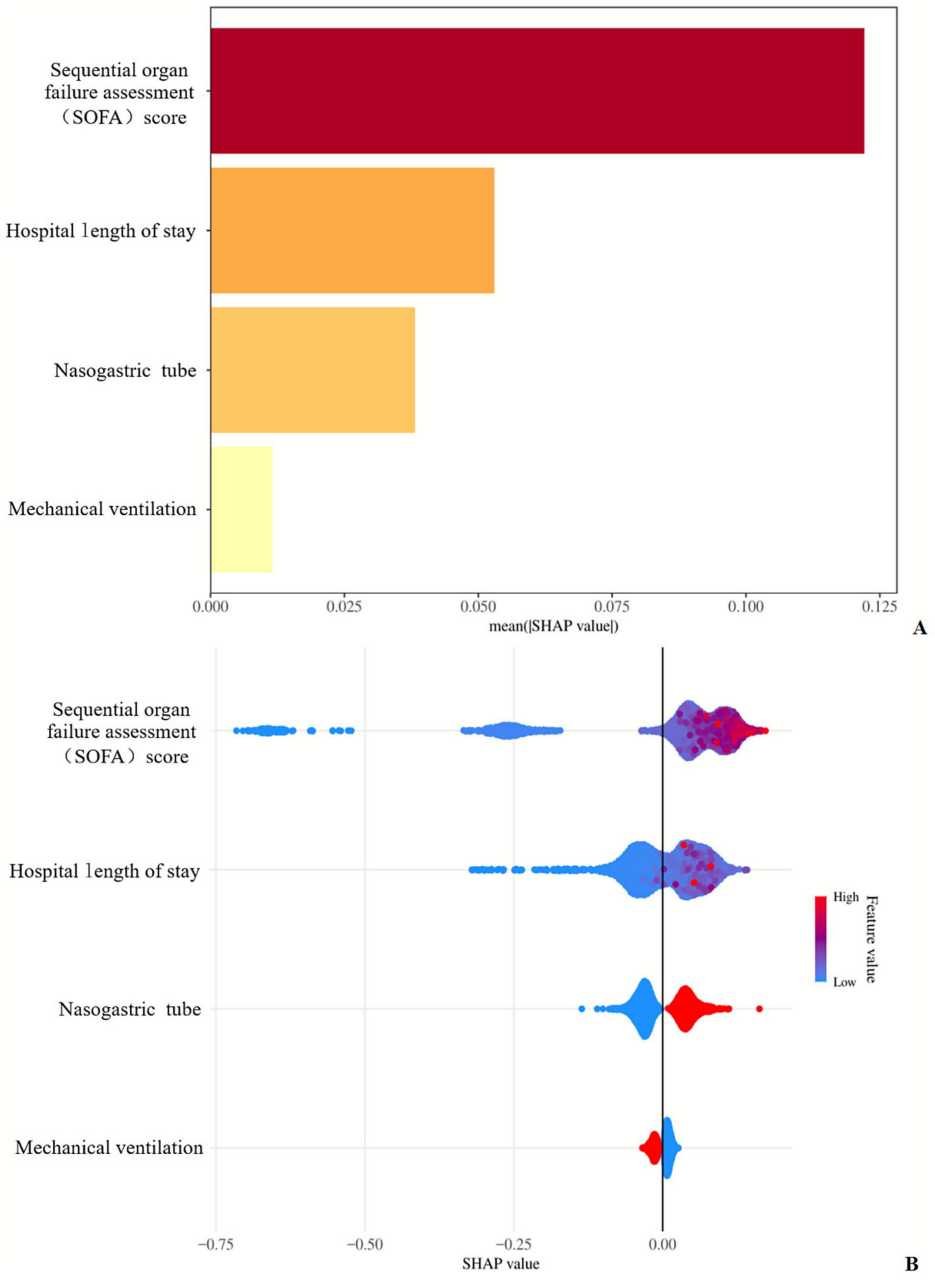
Ranking of predictive factors by weight using the SHAP method.

In the present study, we specifically employed the SHAP (SHapley Additive exPlanations) framework to interpret the predictions generated by the LightGBM model. The summary plot in Figure 6 illustrates the mean absolute SHAP values of each feature across the entire dataset, providing a global-level explanation of which factors the model deems most critical for predictions. Beyond presenting the ranking of feature importance, this plot intuitively visualizes the direction of feature impacts through a color gradient—i.e., whether higher feature values drive the prediction results in a positive or negative direction.

## 4 Discussion

This retrospective study analyzed data from 2,434 stroke patients in the MIMIC-IV Version 3.1 database to evaluate the risk of consciousness disorders, focusing on four key factors: length of hospital stay, mechanical ventilation, nasogastric tube, and Sequential Organ Failure Assessment (SOFA) score. The findings aim to assist healthcare providers in identifying high-risk patients and formulating personalized treatment strategies.

Consciousness disorders may induce complications including dysphagia, pulmonary infections, and dyspnea—risks particularly prominent in ICUs with extensive use of mechanical ventilation. While previous studies have confirmed a significant association between mechanical ventilation and consciousness disorders (18), the present study identifies mechanical ventilation as a protective factor against these disorders in stroke patients: the incidence of consciousness disorders in ventilated patients was only 45% of that in non-ventilated patients. This aligns with prior research showing early ventilation rapidly corrects hypoxemia and alleviates cerebral hypoxia (19), likely by precisely regulating oxygen concentration and ventilation parameters to prevent hypoxia-induced metabolic disturbances and neuronal damage (19) —ultimately reducing the risk of consciousness disorders. Notably, mechanical ventilation lasting over 24 h is classified as prolonged (20), and its long-term use carries risks: it may induce ventilator-associated lung injury or even pulmonary fibrosis (21), which can further cause dyspnea and cerebral hypoxia. These outcomes lead to cerebral cell hypoxia, subsequent cell death, and impaired cerebral function—eventually contributing to consciousness disorders (22).

The ICU inpatients often experience complications (e.g., hypotension, respiratory depression, hallucinations, cerebral hypoperfusion, neurological dysfunction) that prolong hospital stays and elevate the risk of consciousness disorders (23). The present study confirms each additional day of hospitalization increases this risk by a factor of 1.04, consistent with Chen et al. (24), who reported longer stays correlate with poorer prognoses. This may stem from the severe baseline condition of ICU patients: extended hospital stays increase their exposure to multiple pathogens, thereby raising the probability of developing consciousness disorders (25). Thus, clinical teams should prioritize optimizing hospital length of stay by streamlining diagnostic workflows, individualizing therapeutic plans, and enhancing nursing interventions to accelerate patient recovery and reduce consciousness disorder risk.

Regarding nasogastric tube use, the study found ICU patients with such tubes had a 2.47-fold higher incidence of consciousness disorders than those without. Nasogastric tubes are typically used in stroke patients with dysphagia to prevent aspiration and provide nutritional support (26); however, dysphagia itself is linked to brainstem or cortical dysfunction, giving these patients an inherently higher baseline risk of consciousness disorders. Even with tube placement, improper care (e.g., overly rapid feeding, inappropriate patient positioning) can cause regurgitation and aspiration of gastric contents, leading to aspiration pneumonia (27). Inflammatory responses, hypoxemia, or sepsis resulting from pulmonary infections may exacerbate consciousness disorders by impairing cerebral perfusion or inducing systemic inflammatory response syndrome (SIRS). Nursing staff should therefore closely monitor patients’ swallowing function and consider early tube removal once function recovers.

For the SOFA score, each 1-point increase raises the risk of consciousness disorders by 1.60 times. The SOFA score quantifies the severity of organ dysfunction by assessing six organ systems (respiratory, coagulation, hepatic, cardiovascular, central nervous, renal), with each system scored on a 0–4 scale and a total score ranging from 0 to 24 (28); higher scores indicate more severe organ dysfunction or failure (29), and accordingly, a greater likelihood of consciousness disorders. Clinically, it is necessary to promptly perform SOFA scoring for ICU stroke patients and formulate individualized preventive measures in advance based on the results.

In this study, 11 machine learning algorithms were employed to construct risk prediction models for post-stroke consciousness disorders. The results revealed significant differences in performance across algorithms, which reflects the unique characteristics and applicable scenarios of machine learning models in clinical prediction tasks. Among traditional algorithms, logistic regression offers the advantage of strong interpretability (30), however, its AUC values in the training and validation sets were lower than those of most ensemble learning algorithms, indicating that logistic regression has limited ability to fit and generalize complex clinical data. The decision tree model exhibited excellent performance in the training set, but its AUC decreased significantly in the validation set—exposing the limitations of single decision trees, such as susceptibility to overfitting and poor stability.

Among ensemble learning algorithms, the Light Gradient Boosting Machine (LightGBM) demonstrated the optimal comprehensive performance: it achieved the highest AUC in the validation set, with the smallest difference in Brier scores between the training and validation sets. This indicates that through gradient boosting and feature parallel optimization, LightGBM not only avoids the overfitting issue observed in the Random Forest model but also overcomes the limitation of the Elastic Net model—where high sensitivity is accompanied by low specificity. Thus, LightGBM achieves a balance between discriminative ability, stability, and probability calibration. In contrast, the Support Vector Machine (SVM) model performed poorly in both datasets. This is presumably because SVM has insufficient ability to fit the nonlinear relationships in high-dimensional clinical data and is highly sensitive to differences in sample distribution, preventing it from adapting to the complex association patterns of risk factors in this study.

Our Decision Curve Analysis (DCA) results revealed that the model delivers positive net benefits across a clinically relevant risk threshold range of approximately 30 to 90%. This indicates that within this probability spectrum, employing our model to guide clinical decision-making—such as initiating targeted interventions or additional diagnostic workups—yields more favorable patient outcomes compared to the extreme strategies of “treating all patients” or “withholding treatment from all patients.” This specific threshold range holds substantial clinical significance, as it reflects the critical point at which clinicians or patients perceive the benefits of an intervention to outweigh its potential harms. For instance, in the context of the present study, a risk threshold of around 20% might imply that if the model-predicted risk exceeds this cutoff, prophylactic treatment is recommended.

In summary, consciousness disorders in ICU stroke patients are associated with length of hospital stay, mechanical ventilation, nasogastric tube use, and SOFA score. Early assessment of these risk factors and proactive prevention of post-stroke consciousness disorders are therefore of paramount clinical importance. Machine learning algorithms provide diverse tools for predicting the risk of these disorders, and ensemble algorithms such as LightGBM offer distinct advantages in handling multi-factor interactions and improving model generalization. However, the clinical application of such models still requires simplification of operational workflows based on real-world scenarios (e.g., conversion into visual nomograms or online calculators) to enhance their utility in primary care settings.

This study has several limitations. First, its retrospective observational design may inherently introduce selection bias; the direct exclusion of patients with missing variables could lead to outcome bias; and data collection was limited to the early stage of ICU admission, failing to capture the impact of dynamic changes in patients’ conditions on the risk of consciousness disorders. Second, despite the MIMIC-IV database being a large-scale multicenter resource, this study did not conduct external multicenter validation. Third, there are a small number of overlapping indicators between the Glasgow Coma Scale (GCS) score and SOFA score; while the SOFA score is a composite indicator containing more non-overlapping items, data constraints of the MIMIC database prevented the identification of a more suitable alternative indicator. Additionally, research on the subacute phase of stroke was not feasible due to limitations in the available data from the MIMIC database.

Based on the aforementioned limitations, future studies will integrate clinical data to address these issues. Independent multicenter studies are still needed to further verify the generalizability of the established model.

## 5 Conclusion

This study identified length of hospital stay, mechanical ventilation, nasogastric tube use, and SOFA score as independent risk factors for consciousness disorders in ICU stroke patients. Among the 11 machine learning algorithms evaluated, the LightGBM algorithm demonstrated the best performance across three key dimensions: accuracy (assessed by AUC), stability (consistency between training and test set results), and probability calibration (assessed by Brier score). Balancing performance and efficiency, this algorithm can serve as an intuitive, personalized clinical tool to assist clinicians in the timely identification and risk stratification of stroke patients at high risk of consciousness disorders. In turn, this enables the prompt implementation of clinical interventions to reduce the incidence of complications and improve patient outcomes.

## Data Availability

The raw data supporting the conclusions of this article will be made available by the authors, without undue reservation.
