# Supplementary material for: Development and validation of a risk prediction model for consciousness disorders in stroke patients in the intensive care unit (ICU): a retrospective study
Source: Front Med (Lausanne). 2025 Dec 29;12:1668593. doi: 10.3389/fmed.2025.1668593 (PMC12791042; doi:10.3389/fmed.2025.1668593)
Supplement: Supplementary file 1 [file Supplementary_Material.docx]

**Details on Generation of Decision Curve Analysis (DCA) Curves**

All Decision Curve Analysis (DCA) curves in this study were generated using the dcurves R package. The generation process for DCA curves of the training and test sets strictly follows the logic of data standardization, integration of model predictive probabilities, DCA model construction, and visualization optimization. Specific details are as follows:

1. Data Preprocessing and Integration of Predictive Probabilities

1.1 Source of Basic Data

The input data for DCA analysis includes two core components:

True labels (.obs): The target variable status in the classification task (with values "Yes" or "No", corresponding to positive/negative categories), directly derived from the original training set (traindata) and test set (testdata).

Model predictive probabilities: The predictive probabilities of the positive category ("Yes") for each sample, generated by 11 classification models (Logistic Regression, Decision Tree [DT], LASSO, Ridge Regression, Elastic Net [ENet], K-Nearest Neighbors [KNN], LightGBM, Random Forest [RF], XGBoost, Support Vector Machine [SVM], and Single-Hidden-Layer Neural Network [MLP]). These probabilities were extracted from the prediction object of each trained model (core column: .pred_Yes).

1.2 Data Format Conversion

To meet the input requirements of the dcurves::dca() function, the predictive probabilities of each model were integrated into a wide data format through the following steps:

Extract the sample ID (manually generated, corresponding to the row number of the original data), true label (.obs), and positive predictive probability (.pred_Yes) from the prediction results of each model;

Align samples by sample ID and true label, then merge the predictive probabilities of the 11 models as column variables to form a wide data frame (training set: predtrain2; test set: predtest2);

Structure of the wide data frame: id (sample serial number), .obs (true label), Logistic (predictive probability of Logistic Regression), DT (predictive probability of Decision Tree), ... (columns for predictive probabilities of the remaining 9 models).

2. Parameter Settings for DCA Model Construction

2.1 Core Function and Formula

The dcurves::dca() function was used to construct the DCA model. The core formula was defined as follows:

Training set:

dca(as.formula(paste0(".obs ~ ", paste(colnames(predtrain2)[3:ncol(predtrain2)], collapse = " + "))), data = predtrain2)

Test set:

dca(as.formula(paste0(".obs ~ ", paste(colnames(predtest2)[3:ncol(predtest2)], collapse = " + "))), data = predtest2)

Formula interpretation: The true label (.obs) was used as the dependent variable, and the positive predictive probabilities of all models were used as independent variables. All models were included simultaneously for DCA comparison.

2.2 Key Parameter Configuration

Threshold range (thresholds): Set to seq(0, 1, by = 0.01), i.e., 101 decision thresholds were divided from 0 to 1 with a step size of 0.01, covering all potential clinical decision critical points;

Event level (implicit configuration): The positive level of the dependent variable .obs was preprocessed to "second" (i.e., the factor level order was "No" → "Yes"), consistent with the event level used during model training;

Data integrity check: Samples with missing values were automatically filtered out (the original data had been processed via steps such as step_nzv and contained no missing values), ensuring the integrity of the analysis data.

3. Optimization of Curve Visualization

3.1 Plotting Function and Parameters

The dcurves::plot() function was used to visualize the DCA model results, with key parameter settings as follows:

Smoothing (smooth): Set to TRUE; curves were smoothed using Locally Weighted Scatterplot Smoothing (LOESS) to improve readability;

Smoothing span (span): Set to 0.5 to balance the smoothness of the curve and its consistency with the original data trend;

Color configuration (scale_color_manual): A unified color scheme was adopted: the "no intervention" curve was black, the "all intervention" curve was gray, and the curves of the 11 models used a rainbow color series (cols4model <- rainbow(11)), consistent with the color scheme of other model comparison plots in the main text;

Legend position: Set to legend.position = "inside" and legend.justification = c(1,1) (inside the top-right corner) to avoid the legend obscuring the curves;

Grid lines: Panel grid lines were hidden (panel.grid = element_blank()) to highlight the curve trend;

Font: A serif font was uniformly used (family = "serif"), consistent with the font style of figures in the main text.

3.2 Explanation of Abnormal Curve Patterns

If local fluctuations or abnormal patterns appear in the DCA curves of some models, they mainly result from the following reasons (not methodological errors):

Discreteness of model predictive probability distribution: The predictive probabilities of some models (e.g., KNN, SVM) are concentrated in specific intervals, leading to fluctuations in net benefit values within certain threshold ranges;

Sample size and class distribution: Slight imbalance between positive and negative samples in the dataset may cause local abnormalities in net benefit calculations at extreme thresholds (close to 0 or 1);

Trade-off of smoothing parameters: The setting of span = 0.5 balances the smoothing effect and the authenticity of the original data, without excessive smoothing that would distort the curve.

All abnormal patterns are objective reflections of data and model characteristics. No manual modifications were made to the curves, ensuring the authenticity and reproducibility of the analysis results.

4. Dependent Packages and Version Information

Core packages: dcurves v1.4.0 (for DCA model construction and visualization), dplyr v1.1.4 (for data preprocessing), ggplot2 v3.5.1 (for visualization optimization);

Package dependencies: All function calls were based on the package environment loaded in the original code (see the library() calls in the main code), with no additional undeclared dependencies.

The generation of DCA curves in this study can be fully reproduced using the "DCA on traindata" and "DCA on testdata" modules in the original code, with parameter settings completely consistent with the details described above.
